# Dose estimation derived from the exposure to radon, thoron and their progeny in the indoor environment

**DOI:** 10.1038/srep31061

**Published:** 2016-08-08

**Authors:** R. C. Ramola, Mukesh Prasad, Tushar Kandari, Preeti Pant, Peter Bossew, Rosaline Mishra, S. Tokonami

**Affiliations:** 1 Department of Physics, H.N.B. Garhwal University, Badshahi Thaul Campus, Tehri Garhwal - 249 199, India; 2 German Federal Office for Radiation Protection, Berlin, Germany; 3 Radiological Physics and Advisory Division, Bhabha Atomic Research Centre, Mumbai - 400 085, India; 4 Institute of Radiation Emergency Medicine, Hirosaki University, Aomori 036-8564, Japan

## Abstract

The annual exposure to indoor radon, thoron and their progeny imparts a major contribution to inhalation doses received by the public. In this study, we report results of time integrated passive measurements of indoor radon, thoron and their progeny concentrations that were carried out in Garhwal Himalaya with the aim of investigating significant health risk to the dwellers in the region. The measurements were performed using recently developed LR-115 detector based techniques. The experimentally determined values of radon, thoron and their progeny concentrations were used to estimate total annual inhalation dose and annual effective doses. The equilibrium factors for radon and thoron were also determined from the observed data. The estimated value of total annual inhalation dose was found to be 1.8 ± 0.7 mSv/y. The estimated values of the annual effective dose were found to be 1.2 ± 0.5 mSv/y and 0.5 ± 0.3 mSv/y, respectively. The estimated values of radiation doses suggest no important health risk due to exposure of radon, thoron and progeny in the study area. The contribution of indoor thoron and its progeny to total inhalation dose ranges between 13–52% with mean value of 30%. Thus thoron cannot be neglected when assessing radiation doses.

Radon and thoron are produced in the ground by the decay of U-238 and Th-232, respectively. Radon (^222^Rn) with a half-life of 3.825 days tends to concentrate in enclosed spaces such as caves, underground mines and dwellings. Radon (^222^Rn), thoron (^220^Rn) and their progeny are the major contributors to human exposure from natural radiation sources^1^,^2^. Based on epidemiological studies in Europe and North America, the World Health Organization has recognized the exposure to radon and its progeny as second most important cause of lung cancer, after smoking^3^. The inhalation doses resulting from the exposure to radon, thoron and progeny are important quantities to estimate the radiation risk at low dose for epidemiological studies^4^,^5^. Therefore, measurements of radon, thoron and their progeny levels in the indoor environments should be performed using reliable measurement techniques. Although radiological importance of radon and its progeny has been acknowledged a long time ago, thoron had often been neglected due to its short half-life. The contribution of thoron to radiation doses has recently been recognized^6^,^7^,^8^,^9^. Many studies found that thoron can be a significant contributor to the radiation dose in residential buildings in some Asian, European and American countries^7^,^10^,^11^,^12^.

Contrary to the radon and thoron measurements, very few attempts were made for direct measurement of progeny. It has been a usual practice to estimate decay products concentration from gas concentrations using the equilibrium factor approach. However, this approach is not appropriate for estimating thoron decay products concentration from the measured gas concentration due to very short half-life of thoron as compared to its decay products^13^. The consequence of its very short half-life (55 s) is the non-uniformity of thoron concentration in the indoor environment. On the other hand, thoron progeny is usually about uniformly distributed in the room due its longer half-life. Therefore, comparatively very short half-life of thoron as compared to its progeny results in the non-uniformity of thoron equilibrium factor even in the same environment. Hence, it is problematic to estimate thoron progeny concentrations using thoron concentration and an equilibrium factor. Indeed, it is questionable to define an equilibrium factor of thoron because of its physical properties and those of its progeny.

Moreover, since the inhalation doses are predominantly due to progeny of radon and thoron, and not due to the gases, it is important to measure the progeny directly. The progeny of radon and thoron have high diffusivities and ability to stick on the surfaces. Due to this aspect, the freshly formed progeny quickly attach to existing aerosol particles, thereby giving rise to a consecutive activity size distribution. This distribution is broadly classified into two groups, namely, the fine/unattached fraction (~2 nm diameter) and the coarse/attached fraction (~150 nm)^14^. The attached fraction is likely to pass the upper respiratory tract and to leave alveoli during exhalation; a major part of the unattached fraction also passes the upper respiratory tract but is deposited in the alveoli and then is subjected to somatic transport processes. The unattached fraction is absorbed by blood at faster rate compared to the attached fraction^15^. The unattached fraction is predominantly responsible for radiation dose received by the target cells in the bronchial epithelium^16^. Thus, it is important to measure both the size modes for accurate dose assessment.

Although the exposure to indoor radon, thoron and their progeny is considered a major problem in cold climate countries, but not a serious concern in India because of usually higher ventilation rates, there is a need to identify the levels of radon, thoron and their progeny in the Indian dwellings. India comprises of different climatic regions (zones). Some parts of the country are extremely hot whereas some other parts are extremely cold. The study area in the present investigation is one of the cold climate regions of India, with towns and settlements lying in Garhwal Himalaya at altitudes of up to 3000 m above sea level.

In the present study, the measurements of radon, thoron and their progeny concentrations were performed in 122 houses of Yamuna, Tons and Kedar valleys of Garhwal Himalaya. The radon and thoron concentrations were measured with LR-115 detector based single entry pin-hole dosimeter technique. The attached and unattached progeny concentrations of radon and thoron were measured separately. The measurements of total (attached + unattached) and unattached progeny concentrations were performed using DRPS/DTPS in bare mode and wire mesh capped DRPS/DTPS, respectively. The unattached progeny concentrations were calculated by simply subtracting the attached progeny concentrations from total progeny (attached + unattached) concentrations. The unattached fractions of radon and thoron progeny were estimated as the ratio of the unattached progeny concentrations to the total (attached + unattached) progeny concentrations. The equilibrium factors for radon and its progeny and for thoron and its progeny were calculated for the individual dwellings. Finally, the radiation doses (total annual inhalation dose and annual effective doses) were estimated from the measured values of activity concentrations.

## Materials and Methods

### Study area and selection of dwellings

**Figure 1:**
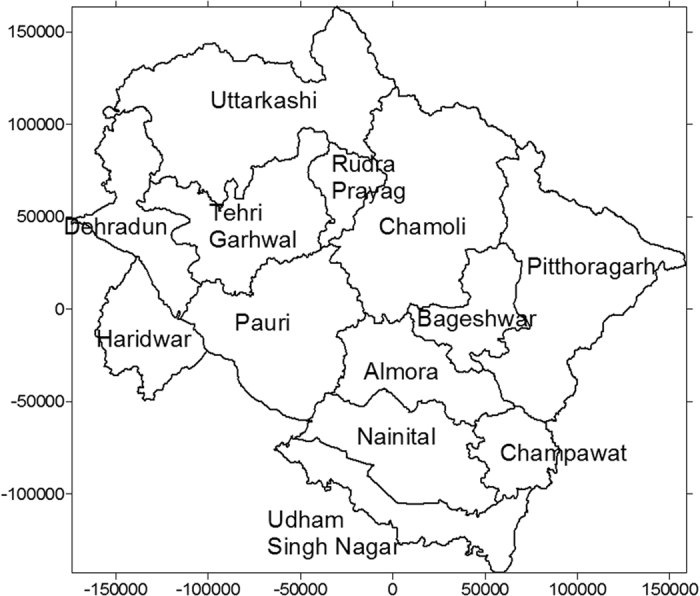
Shape of Uttarakhand state of India in geographical coordinates. Map was created with Golden Software Surfer version 8 (www.goldensoftware.com).

**Figure 2:**
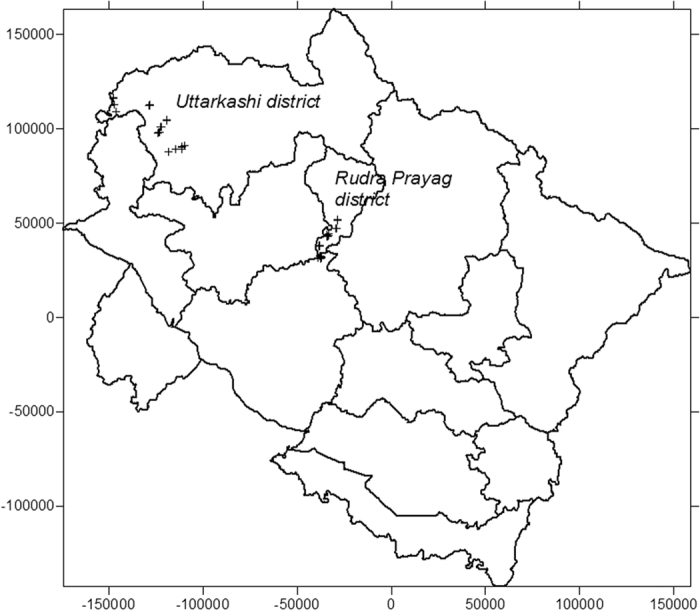
Map of Uttarakhand showing the sampling sites covering Uttarkashi and Rudraprayag districts. Map was created with Golden Software Surfer version 8 (www.goldensoftware.com).

**Figure 3:**
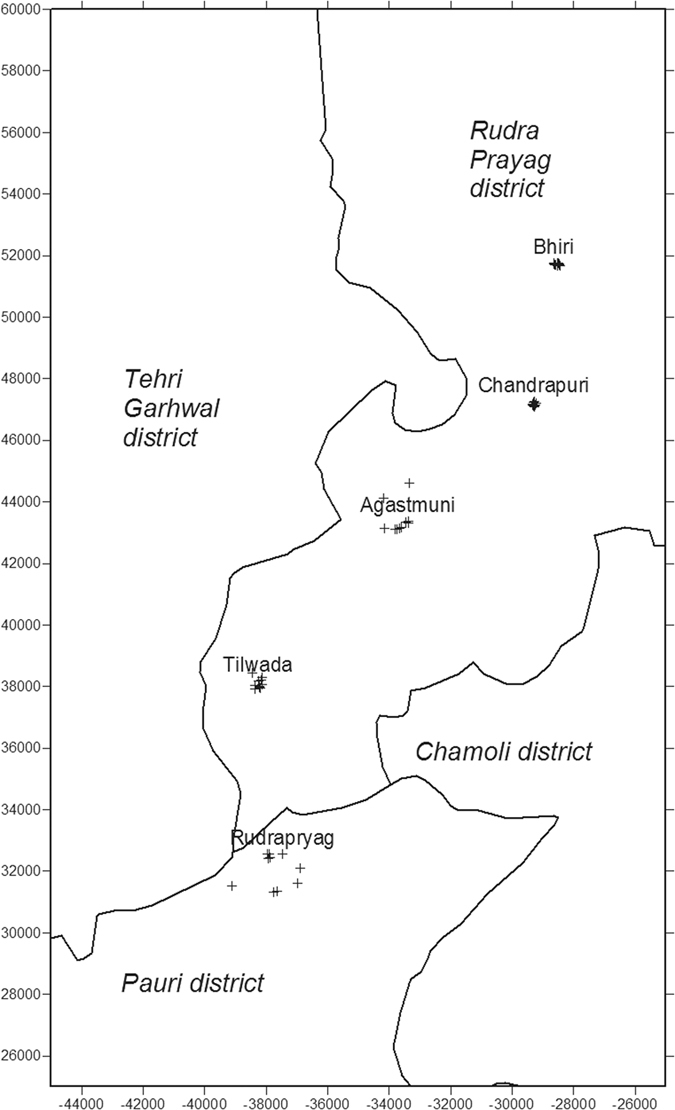
Map showing the sampling sites in Kedar valley of Rudraprayag district. Map was created with Golden Software Surfer version 8 (www.goldensoftware.com).

**Figure 4:**
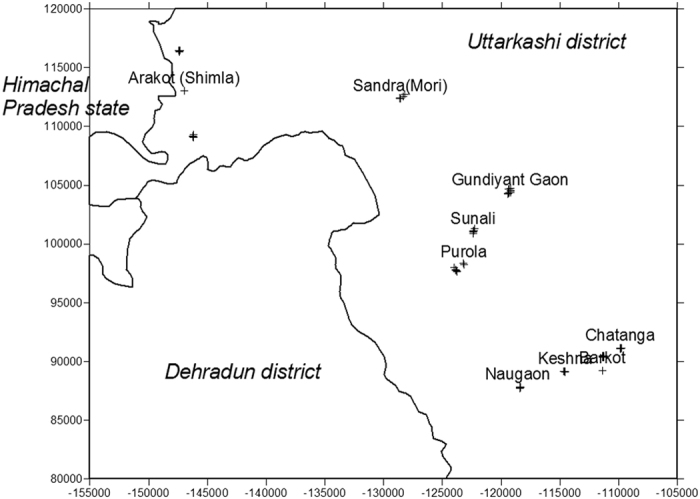
Map showing the sampling sites in Yamuna and Tons valleys of Uttarkashi district. Map was created with Golden Software Surfer version 8 (www.goldensoftware.com).

The study area is located in the Yamuna, Tons and Kedar valleys of Garhwal Himalaya, India. The geographical maps of the study areas are shown in the Figs 1, 2, 3, 4. The maps were prepared with Surfer software version 8 (http://www.goldensoftware.com) using Lambert Conformal Conical (LCC) according to NNRMS^17^, where the LCC parameters for the State of Uttarakhand (Uttaranchal) are given in Table 4.2, Page 30. The transform formulae^18^ can be found in Snyder (1987; p. 104 ff., eqns. 14-1 to 15-3). The zero point was set deliberately. No false Easting and Northing was applied. Earth was assumed spherical for simplicity with radius 6371 km. Shape files of administrative borders were retrieved from http://biogeo.ucdavis.edu/data/diva/adm/IND_adm.zip (22 June 2015).

The Yamuna and Tons valleys lie in the Rawain and Jonsar Bhabhar regions of Uttarkashi district of Uttarakhand state of India, respectively. The Kedar valley lies in the Rudraprayag district of Uttarakhand state of India. The internationally known Kedarnath temple situated in Kedarnath town (at the top of Kedar valley) is an important and attractive place for pilgrimage and trekking in the north of the Kedar valley. Usually, several thousand pilgrims visit the temple every year during May to October. The study areas suffered extensive destruction during June 2013 from flash-floods caused by torrential rains in Uttarakhand state of India. The surrounding area near Kedar valley was destroyed by the flood. Thousands of people were killed and thousands of others (mostly pilgrims) were reported missing or stranded due to landslides in the nearby areas. It was reported that previously uncollected bodies were still being found one year after the tragedy. Since this destruction brought a huge change in the local surface geology of the study area, an attempt has been made by the authors to investigate the radiation risk due to the exposure of indoor radon, thoron and their progeny in these areas. The study area is scattered in remote mountainous terrain. The selection of dwellings in the study areas was done keeping in mind to cover as much of the area of the region as logistically feasible. The efforts were made to select the houses with all influencing factors represented as much as achievable, such as building materials (cement, mud, stone, wood, etc.), traditional and new houses, ventilation conditions and geographical location. The measurements were made in 122 houses. However, 42 houses were selected for study of seasonal variations in radon, thoron and progeny. The main aim is to cover different types of houses, so that it can be used as representative values of radon and thoron levels in the area surveyed.

### Measurements of indoor ^222^Rn/^220^Rn concentrations

**Figure 5:**
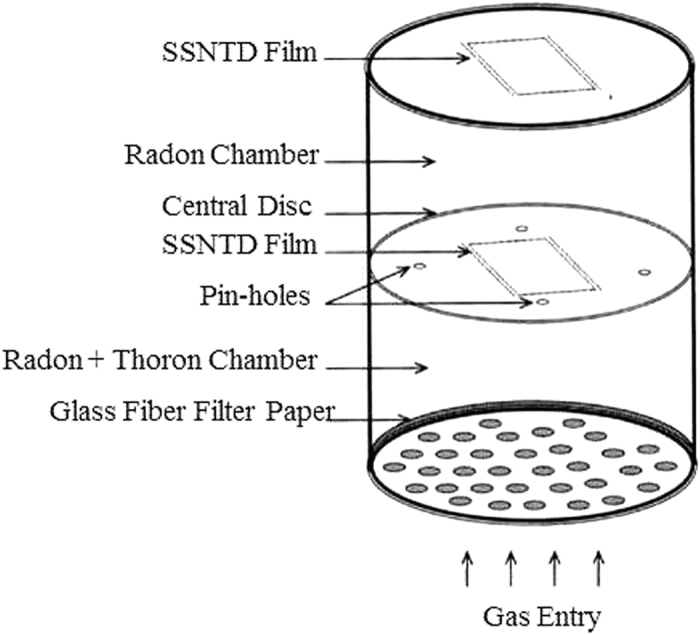
Schematic diagram of single entry pin-hole dosimeter.

Measurements of ^222^Rn and ^220^Rn were carried out by LR-115 Type II detector based pin-hole dosimeter technique. The dosimeter is a cylindrical plastic chamber and consists of two equal compartments separated by a central disc, each compartment having a length of 4.1 cm and radius 3.1 cm. Four pin-holes, each having a length of 2 mm and 1 mm diameter are made on this circular disk in order to discriminate ^220^Rn. The dosimeter has only one entrance through which the gas enters the first chamber namely “radon + thoron” compartment through a 0.56 μm glass fiber filter paper and subsequently diffuses to second chamber called “radon” chamber, essentially cutting off the entry of thoron into this chamber because of its very short half-life of 55.6 seconds compared to that of radon (3.825 days). The pinholes act as diffusion barrier. The LR-115 detector films are fixed at the end of each compartment. The device has been calibrated in a laboratory facility at Bhabha Atomic Research Centre, Mumbai in order to find the correlation between tracks registered on the detector films and the concentration of radon/thoron^19^. The alpha emissions from radon and thoron produce the tracks on LR-115 detector film placed at the end of first chamber while as there is only radon and not thoron in the second chamber, the tracks are registered on the LR-115 detector film placed at the top of this chamber due to the alpha emissions of radon only. The dosimeters were suspended indoor overhead on the ceiling at the minimum height of 1.5 m from the ground and at least 10 cm away from any wall surface for a period of about 3 months. The distance from the wall was chosen because it is assumed that most thoron is exhaled from building materials and due its short half-life (55 sec) concentrates close to wall surfaces. The schematic diagram of the single entry pin-hole dosimeter is shown in the Fig. 5.

### Measurement of ^222^Rn/^220^Rn progeny

LR-115 Type II (12 μm cellulose nitrate film coated on a 100 μm thick polyester base) solid state nuclear track detector deposition based direct progeny sensor technique has been used for the measurement of ^222^Rn and ^220^Rn progeny. Direct progeny sensors are made of passive detectors (LR-115 type II) mounted with absorbers of appropriate thickness. For thoron (^220^Rn) progeny, the absorber is aluminum Mylar of 50 μm thickness, which selectively records the tracks due to alpha particles emitted from ^212^Po (α energy 8.78 MeV). For radon (^222^Rn) progeny, the absorber is a combination of aluminized Mylar (25 μm) and cellulose nitrate film (12 μm) of effective thickness 37 μm, which mainly detects alpha particles emitted from ^214^Po (α energy 7.69 MeV) and ^212^Po (α energy 8.78 MeV). The sensor, which is used for the detection of thoron (^220^Rn) progeny, is known as Direct Thoron Progeny Sensor (DTPS) and the sensor which is used for the detection of radon (^222^Rn) progeny is known as Direct Radon Progeny Sensor (DRPS). The basic principle of the operation of these sensors is that the LR-115 detector detects the alpha particles emitted from the deposited progeny atoms. In calculating the progeny concentrations, the track density obtained using DTPS can be used directly to calculate the equilibrium equivalent thoron concentration (EETC), since the comparatively larger thickness (50 μm) of the absorber used in DTPS does not allow radon progeny to pass through it and hence there is no interference of radon progeny to thoron progeny. In case of equilibrium equivalent radon concentration (EERC), since α energy of ^212^Po (thoron progeny) is higher than that of ^214^Po (radon progeny), the alpha particles emitted from both radon progeny as well as from thoron progeny pass through the absorber (37 μm) used in the DRPS and hence to calculate EERC, the tracks produced by the thoron progeny are eliminated as calculated from DTPS, using the equation^20^:


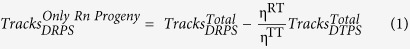


where, η^RT^ and η^TT^ are the track registration efficiencies of thoron progeny in DRPS and that in DTPS, respectively, 
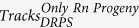
 is the tracks density recorded on DRPS due to only radon progeny and 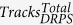 and 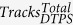 are the abbreviations for total track density recorded on DRPS and DTPS, respectively.

**Figure 6:**
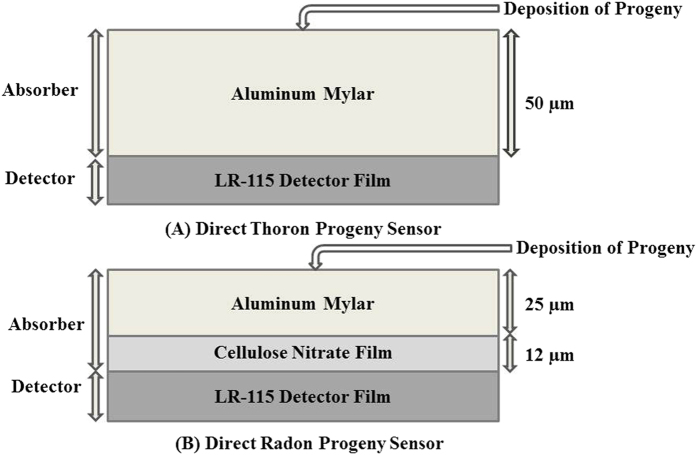
Schematic diagram of direct progeny sensors in bare mode.

Formulae used to calculate EETC and EERC^20^,^21^ are given as follows:


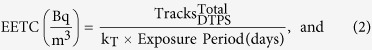



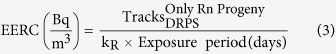


where, k_T_ and k_R_ are calibration factors (sensitivity factors) for DTPS and DRPS, respectively. The values of sensitivity factors for DTPS and DRPS in natural environment have been calculated by Mishra *et al.*^13^ to be equal to 0.94 Tracks cm^−2^ d^−1^/EETC (Bq m^−3^) for DTPS and 0.09 Tracks cm^−2^ d^−1^/EERC (Bq m^−3^) for DRPS. The schematic diagram of the direct progeny sensing system is given in the Fig. 6.

### Measurements of attached/unattached radon and thoron progeny concentrations

**Figure 7:**
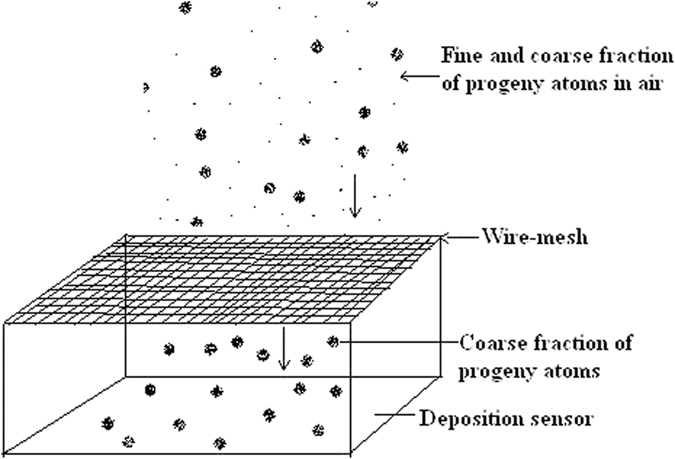
Schematic diagram of wire-mesh capped direct progeny sensors.

Wire-mesh Capped DTPS/DRPS was used in Passive Mode for measurements of attached and unattached radon and thoron progeny concentration. Wire-mesh capped DTPS/DRPS (Fig. 7) consists of DTPS/DRPS capped with a 200 mesh type wire-screen, such that the whole system is actually used as a fine fraction separator, or in other words, coarse fraction detector. The unattached fraction of the progeny atoms are trapped on the wire-mesh, and only the attached fraction of the progeny atoms get deposited on the DTPS/DRPS. The alpha particles deposited on the capped DTPS/DRPS is a measure of attached fraction of progeny activity concentration. The loaded wire-mesh capped DTPS/DRPS can be installed in the same way as the bare mode DTPS/DRPS. The measurement technique is explained elsewhere in details^14^.

The detectors exposed for about three months are retrieved and sealed in doubly aluminized bags until further analysis. Then the DTPS/DRPS are removed from the wire-mesh capped system and subjected to the chemical etching followed by counting the tracks using spark counter as in case of bare mode DTPS/DRPS. The background tracks in the detector during the detector shelf life as well as during handling should be subtracted from the observed track density. The tracks registered in the capped DTPS/DRPS can be related to the attached fraction of EETC by using a sensitivity factor of 0.33 (Tracks.cm^−2^.d^−1^)/(Bq.m^−3^). Similarly the sensitivity factor of DRPS in wire-mesh capped mode is 0.04 (Tracks.cm^−2^ d^−1^)/(Bq m^−3^).

### Chemical processing and analysis of LR-115

The exposed LR-115 detector films were etched in an etching bath using 2.5 N NaOH solutions at 60 °C temperature for 90 minutes without stirring. The tracks recorded on the films were then counted using spark counter, which is an electronic counter operating at high voltage. The resulting average track densities were converted into radon, thoron and progeny concentrations using calibration factors discussed above.

### Estimation of equilibrium factor

In the past, it has been a usual practice to estimate the radiation dose quantities due to exposure of radon, thoron and its progeny using worldwide assumed value (0.4) of equilibrium factor for radon and its progeny. However, the equilibrium factor depends largely on the environmental conditions such as hours and modes of ventilation, humidity, time, place etc.^22^,^23^,^24^,^25^,^26^. In contrast, thoron equilibrium factor varies significantly even for the same environment due to wide variation of thoron concentration arising from its short lived nature. The very short half-life of thoron results in non-uniformity of thoron concentration in the indoor environment. Hence it is not advisable to estimate radiation doses of thoron using the gas concentration and an equilibrium factor which depends on the sampling protocol, in addition to environmental factors as for radon. In the present study, direct measurements of the decay products concentrations and gas concentrations were carried out by using direct progeny sensors and pin-hole dosimeter techniques, respectively. The equilibrium factor for radon and its progeny and thoron and its progeny were then simply calculated for individual dwellings by using the following expressions:


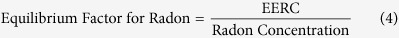



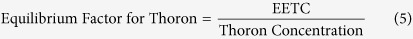


where, the quantities radon concentration, thoron concentrations EERC and EETC represent the arithmetic means (AM) over the measurement period (about 3 months). We want to mention that there is also an alternative definition of equilibrium factor, namely the mean over ratios of short term measurements. Note that in general, the ratio according our definition; AM (progeny)/AM (gas) is not equal to AM (progeny/gas). Using the geometrical means (GM) instead, the two definitions would be identical. This has also been discussed by Mishra *et al.*^27^

### Estimation of radiation doses

The measured values of activity concentrations of radon, thoron and their progeny in the study area have been used to estimate the total annual inhalation dose and annual effective doses due to the exposure of these nuclei.

### Estimation of total annual inhalation dose

The total annual inhalation dose due to exposure of indoor radon, thoron and their progeny has been calculated by using the relation given by UNSCEAR^28^.


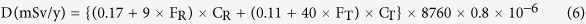


where, F_R_ and F_T_ are the equilibrium factors for radon and its progeny and thoron and its progeny, respectively. C_R_ and C_T_ are the radon and thoron concentrations in Bq/m^3^, respectively. The quantities 0.17 and 9 are dose conversion factors for radon and its progeny concentrations, respectively while the quantities 0.11 and 40 are the dose conversion factors for thoron and its progeny concentrations in nSv units, respectively^28^. The indoor occupancy factor was assumed 0.8 as standard for the study area^29^. The multiplication factor 10^−6^ is used to convert the nSv units into mSv units. Since the equilibrium factors vary with environmental factors, these factors have been estimated independently for individual dwellings separately. Note that with new radon dosimetry currently elaborated by the ICRP, the doses could be a factor 2 to 3 higher^30^,^31^.

### Estimation of annual effective doses

The annual effective dose due to exposure of radon progeny has been calculated by the relation given by UNSCEAR^28^:


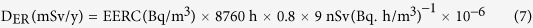


Similarly, the effective dose due to exposure of thoron progeny may be calculated by the relation:


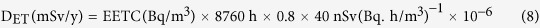


where, EERC and EETC are the equilibrium equivalent concentrations of radon and thoron, respectively, in the houses of the study area. The numerical quantity 0.8 is the annual indoor occupancy factor, 9 and 40 are the dose conversion factors for radon and thoron progeny in nSv units, respectively^28^,^29^. The multiplication factor 10^−6^ is used to convert the nSv units into mSv units.

### Study of seasonal variations

In order to study the effect of environmental parameters such as ventilation conditions, temperature, humidity etc. on indoor radon, thoron and progeny concentrations, the measurements of seasonal variations were performed for three seasons of a year, namely: winter (November–February), summer (March–June) and rainy (July–October) seasons. However, in some cases, the detector could not be replaced exactly 120 days after their deployment due to logistic reasons. In such cases, slight deviations of upto 10 days occurred. Therefore, an attempt has been made to calculate the weighted average of the species of interest over the exposure days for the particular season. The study of seasonal variations was carried out in 42 houses of the study area out of 122 houses in which the measurements were done for first (rainy) season.

### House type comparison

An attempt was made to investigate the effect of building materials and design of houses on radon, thoron and progeny levels. For this purpose, the measurements were carried out in different 62 houses. These 62 houses were characterized into 3 different categories, namely: mud houses, cemented houses and wooden houses. The mud houses are of older style and are made of local stones and mud. The roofs of the houses are made with slates. These houses with small dimensions and having one door and one small window or without window are poorly ventilated. The cemented houses are made of cement, stones and bricks. The roofs are made with cement, concrete and iron bars and the floors are made with cement and concrete in such type of houses. The wooden houses have traditional design with second stories. The principal building material of wooden houses is wood. The rooms of the houses are comparatively of small dimensions.

### Estimating missing values

In order to estimate the total annual inhalation dose and annual effective doses, the annual average concentrations of radon, thoron and progeny are required. However, in some cases, results for one season were missing due to lost of detectors or logistical difficulties. In order to estimate the annual mean in such cases, we applied a method inspired by Stojanowska *et al.*^32^ Suppose there are N cases, out of which if K cases of season 1 (without loss of generality) are missing. For the remaining (N–K) *complete* cases, the *complete annual mean* is calculated as weighted arithmetic mean (wAM), taking exposure periods (t_i_) in each season as weighting factor. The *complete mean* is calculated using the relation:


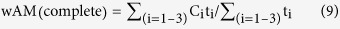


where, C_i_ is the measured concentration in season (i). For the same cases, the incomplete mean is calculated leaving the data for season 1. The *incomplete mean* is calculated using the relation:


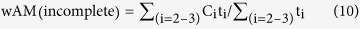


Then, least square linear fit is performed by regressing wAM (complete) as dependent variable against wAM (incomplete) as independent variable. This yields a model of the annual mean as function of the incomplete mean. This model is applied to the K incomplete cases, resulting in *estimates* of the annual mean for these cases. An estimate of the concentration in the missing season can be gained by inverting the formula of wAM (complete) with respect to C_1_, and t_1_ = 365 − t_2_ − t_3_ − t_4_. In practice, in different cases different seasonal concentrations (C_i_) are missing. Thus, the method has to be repeated accordingly, with different wAM (incomplete) with respect to the missing season. To be sure, the method only works if regression turns out significant, i.e. with high R^2^ and low p-value (<0.05).

## Results and Discussion

### Descriptive statistics of radon, thoron and progeny in the houses of study area for rainy season

**Figure 8:**
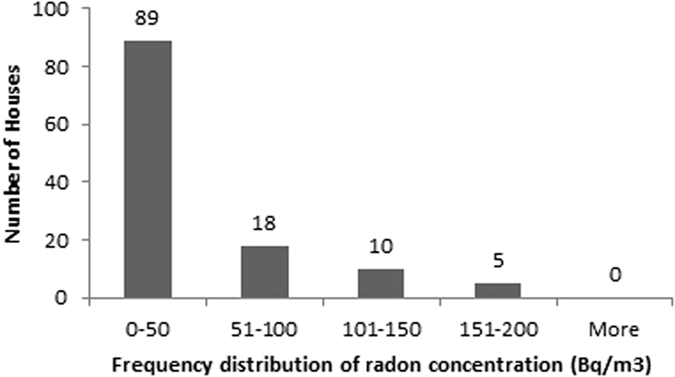
Frequency distribution of radon concentrations in 122 houses of Garhwal Himalaya for rainy season.

**Figure 9:**
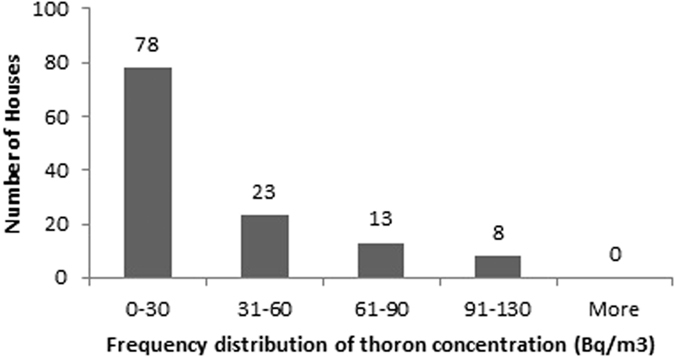
Frequency distribution of thoron concentrations in 122 houses of Garhwal Himalaya for rainy season.

**Figure 10:**
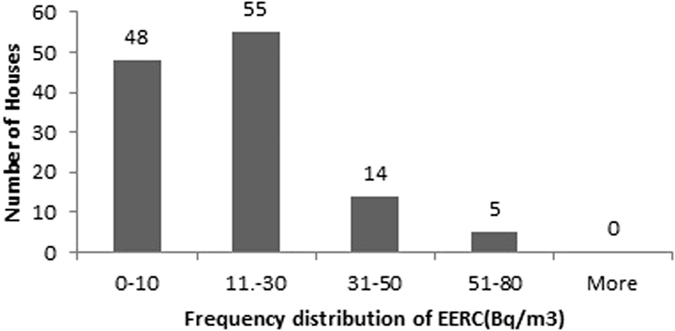
Frequency distribution of EERC in 122 houses of Garhwal Himalaya for rainy season.

**Figure 11:**
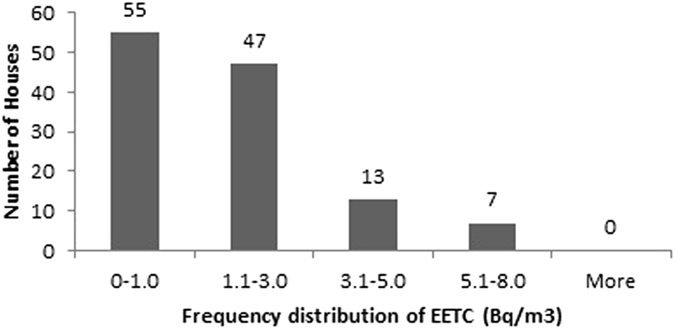
Frequency distribution of EETC in 122 houses of Garhwal Himalaya for rainy season.

The descriptive statistics of the findings obtained for radon, thoron and progeny for rainy season in 122 houses of Garhwal Himalaya is shown in the Table 1. The measured values of radon and thoron concentrations have been found to vary from 4 ± 1 Bq/m^3^ to 198 ± 9 Bq/m^3^ with a mean value of 41 ± 44 Bq/m^3^ and 1 ± 0.7 Bq/m^3^ to 127 ± 8 Bq/m^3^ with a mean value of 33 ± 30 Bq/m^3^, respectively. The measured values of equilibrium equivalent radon concentration (EERC) and equilibrium equivalent thoron concentration (EETC) have been found to vary from 1 ± 0.2 Bq/m^3^ to 76 ± 3 Bq/m^3^ with a mean value of 17 ± 14 Bq/m^3^ and 0.1 ± 0.03 Bq/m^3^ to 7 ± 0.3 Bq/m^3^ with a mean value of 2 ± 1 Bq/m^3^, respectively. Frequency distribution of radon, thoron and progeny concentrations in 122 houses of the study area is shown in the Figs 8, 9, 10, 11. Out of 122 houses, radon concentration has been found ≤50 Bq/m^3^ in 89 houses, thoron concentration has been found ≤30 Bq/m^3^ in the 78 houses, EERC has been found to be in the range 11 Bq/m^3^–30 Bq/m^3^ in 55 houses and EETC has been found ≤1 Bq/m^3^ in 55 houses.

As discussed earlier, out of 122 houses in which the sampling was done for first (rainy) season, the measurements were repeated in 42 houses for two more seasons (winter and summer seasons) of a year in order to have the seasonal variations of radon, thoron and progeny concentrations. The ratio of an activity concentration for rainy season to annual average concentrations of the corresponding species obtained from these 42 houses was used to estimate the annual average concentration for 122 houses sampled for rainy season.

The estimated values of annual mean concentrations of radon, thoron, radon progeny (EERC) and thoron progeny (EETC) were found to be 55 ± 59 Bq/m^3^, 64 ± 48 Bq/m^3^, 23 ± 21 Bq/m^3^ and 2 ± 1.9 Bq/m^3^, respectively. The estimated values of annual average concentrations of radon (55 Bq/m^3^) and thoron (64 Bq/m^3^) were found to be higher than the global average values of 40 Bq/m^3^ and 10 Bq/m^3^ as well as the national average values of 42 Bq/m^3^ and 12.2 Bq/m^3^ of radon and thoron concentrations^20^,^28^.

### Dependence of radon, thoron and progeny concentrations on seasons

The variations of indoor radon, thoron concentrations, EERC (attached + unattached), EERC (attached), EERC (unattached), unattached fraction of radon progeny, EETC (attached + unattached), EETC (attached), EETC (unattached), unattached fraction of thoron progeny and equilibrium factors for radon and thoron with three seasons of a year are given in the Table 2. It should be noted here that the results of radon, thoron and their progeny levels for rainy season represented in the Table 1 are not the same as those for rainy season represented in the Table 2. This is because of the fact that Table 1 represents the results for 122 houses for rainy season while Table 2 represents the results for 42 houses.

The average value of radon concentration was found to be minimum in the summer season and maximum in the winter season. On the other hand, the average value of thoron concentration was found minimum in the rainy season and maximum in the winter season. The annual average values of EERC and EETC (attached + unattached) were found to be minimum in the summer season and maximum in the winter season. The higher values of radon concentration in winter may be ascribed to the fact that during the colder months (winter season), inhabitants of the study area used to keep the doors and windows closed which results in poor ventilation between indoor and outdoor environments. On the other hand, comparatively lower levels of radon concentrations in the summer season may be attributed to the fact that due to the higher average temperature in the study area during summer season there is higher air exchange rate between indoor and outdoor environment which results in the good ventilation between indoor and outdoor environments. These types of seasonal variations have also been observed by other authors in Garhwal Himalaya region^33^,^34^.

**Figure 12:**
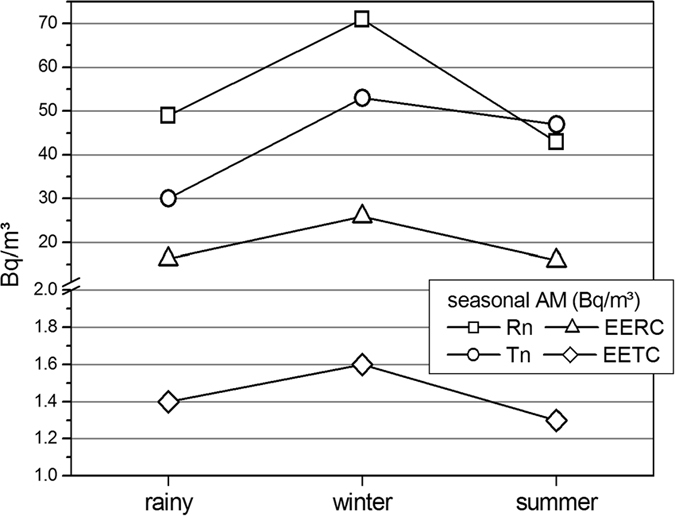
Seasonal dependence radon, thoron and progeny concentrations (Bq/m^3^).

However, the ventilation conditions do not influence the indoor thoron concentration as in case of radon concentration because of its very short half-life (55.6 seconds). Due to this thoron has very short diffusion length (<10 cm) and it cannot travel longer distances before it decays. Nevertheless, the average thoron concentrations have been found to be highest in the winter season as in case of radon concentration. The comparatively lower average value of thoron concentration in the rainy season may be because of the fact that during the rainy season, the capillaries of soil are mostly occupied by the water and thoron cannot escape easily from these capillaries due to its very short half-life. Such a dependence of thoron concentration in rainy season was reported by Ramola^35^ in the past. The seasonal dependence of radon, thoron and their EECs is represented in the Fig. 12.

### Attached/unattached concentrations and unattached fractions for radon and thoron progeny

The attached/unattached concentrations and unattached fractions of radon and thoron progeny are represented in Table 2. It has been observed that 86% of the total annual mean radon progeny concentration is attached and 14% is unattached. On the other hand, 79% of the total annual mean thoron progeny is attached and 21% is unattached. The annual average values of unattached fractions of radon and thoron progeny have been found to 0.12 ± 0.07 and 0.19 ± 0.09, respectively. It can be seen that unattached fraction of thoron progeny is higher than that of the radon progeny.

**Figure 13:**
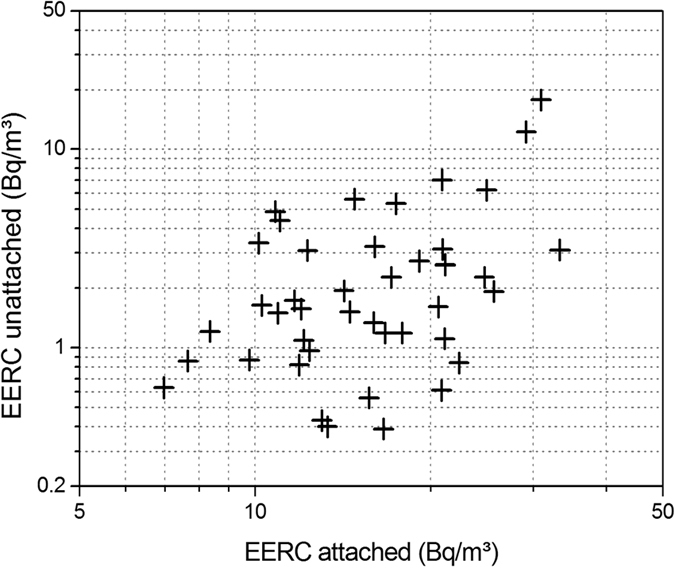
Correlation between attached and unattached EERC (Bq/m^3^).

**Figure 14:**
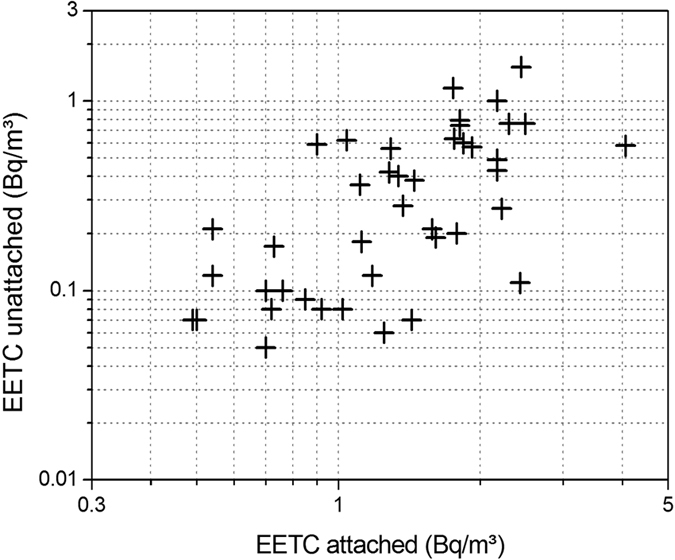
Correlation between attached and unattached EETC (Bq/m^3^).

From Figs 13 and 14, it can be seen that the concentrations of the attached and unattached fractions are positively correlated, although only weakly, with Pearson correlations R^2^ = 0.28 and 0.31, for radon and thoron, respectively (significant with p < 0.01 in both cases).

**Figure 15:**
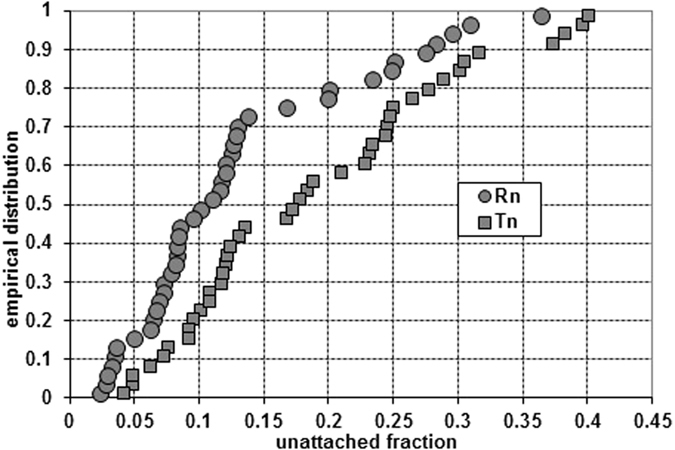
The shapes of histograms or the empirical distribution function for attached and unattached fractions.

We estimate the unattached fractions as medians of (unattached)/(total), resulting in 0.11 (0.030 to 0.29) and 0.18 (0.050 to 0.38) for radon and thoron, respectively (In brackets: 5–95% confidence intervals). The differences between medians and means are significant at p = 0.05 (t-test, Mann-Whitney and Kolmogorov-Smirnov tests). The shapes of histograms or of the empirical distribution function suggest that the distributions might be (at least) bi-, rather than unimodal (Fig. 15).

### Dependence of radon, thoron and progeny on house type

Sixty two houses of the study area were selected for house type comparison and these houses were characterized into 3 categories, namely: cemented houses, mud houses and wooden houses. The measured values of indoor radon, thoron and progeny concentrations in different types of houses are shown in the Tables 3 and 4.

It has been seen that the average values of radon, thoron and their progeny concentrations were found higher in the ground floors as compared to the first floors in all the types of houses. This is attributed to the fact that soil underneath ground along with the building material is the major source of indoor radon and thoron in the ground floor. On the other hand in the first floor, the building material may contribute relatively more to radon and be the only source of thoron.

Further, if we compare the average radon and thoron concentrations in the ground floors of all the three types of houses, we can see that the average radon and thoron concentrations are highest in the mud houses and lowest in the wooden houses. Likewise, average values of EERC and EETC were found highest in the mud houses and lowest in the cemented houses if we compare the activity concentrations in the ground floors of the of houses. This may be due to the fact that in the cemented houses there is a coating of cement on the floor which attenuates the entry of radon and thoron into indoor environment resulting in low concentration in cemented houses. On the other hand, as there is no such coating on the floor in the mud houses, soil pores are open on the floor and radon/thoron can enter from the soil to indoor environment easily. Such relations have also been observed in the past for Garhwal Himalaya^36^. The emanation from the ground surface and from the building materials of mud houses results in the high value of radon and thoron in the room^36^. The comparatively lower levels of radon and thoron concentrations in the wooden houses as compared to cemented houses and mud houses may be due to the fact that wooden walls of such houses are not the source of radon and thoron.

### Estimation of equilibrium factors for radon and thoron

**Figure 16:**
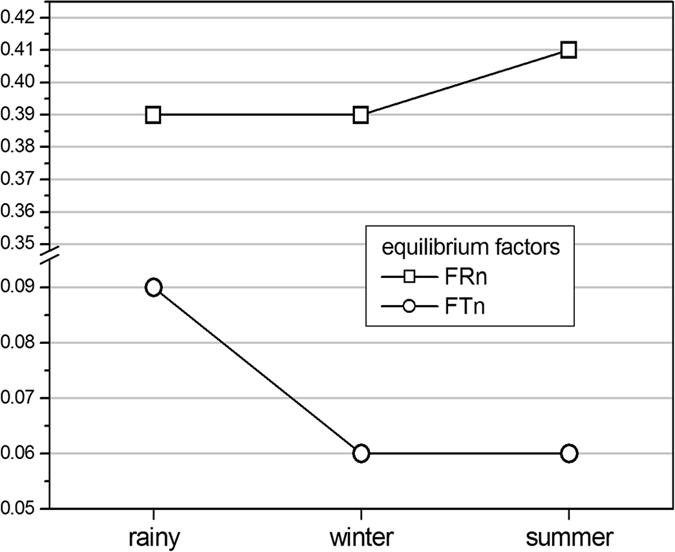
Seasonal dependence of equilibrium factors for radon (F_RN_) and thoron (F_Tn_).

The annual average value of equilibrium factor for radon and its progeny and thoron and its progeny were found to vary from 0.10 to 0.62 with an average of 0.4 ± 0.13 and 0.02 to 0.17 with an average of 0.07 ± 0.04 respectively. The seasonal variations of equilibrium factors for radon and thoron are graphically represented in Fig. 16. The annual average value of equilibrium factor for radon and its progeny has been found to be in good agreement with its globally assumed value (0.4) as reported in UNSCEAR^28^ and recently calculated value (0.42) for Garhwal Himalaya^37^. However, this factor has been found to be higher than the previously determined value (0.28) of equilibrium factor for radon and its progeny for Garhwal Himalaya^24^. The annual average value (0.07) of equilibrium factor for thoron and its progeny has been found to be lower than the globally assumed value (0.1) as reported in UNSCEAR^38^ and previously determined value (0.09) for Garhwal Himalaya^24^. However, this factor has been found to be in good agreement with the recently determined value of equilibrium factor for thoron and its progeny for Garhwal Himalaya^34^. The deviation from previously reported value of equilibrium factor may be due to use of improved methodology. Moreover, the selection of houses may also have produced the different results. This study indicates that the values of equilibrium factors for radon and thoron are reproducible in the study area with recently developed pin-hole dosimeter and DTPS/DRPS techniques.

### Estimation of radiation doses

The measured values of annual average concentrations of radon, thoron and progeny in 42 houses of Garhwal Himalaya were used for estimating the radiation dose received by the population of the study area. The total annual inhalation dose due to the exposure of indoor radon, thoron and their progeny in study area has been found to vary from 0.8 mSv/y to 4.6 mSv/y with an arithmetic mean value of 1.8 ± 0.7 mSv/y. The annual effective dose due to the exposure of radon and its progeny in the study area has been found to vary from 0.5 mSv/h to 3.1 mSv/h with an average of 1.2 ± 0.5 mSv/h. The annual effective dose from the exposure to thoron and its progeny in the study area has been found to vary from 0.2 mSv/h to 1.3 mSv/h with an average of 0.5 ± 0.3 mSv/h, contributing about 1/3^rd^ to the total dose. The geometric mean values of total annual inhalation dose, annual effective dose due to radon and its progeny and annual effective dose due to thoron and its progeny in the houses of the study area was found to be 1.7 mSv/y, 1.1 mSv/y and 0.5 mSv/y, respectively.

**Figure 17:**
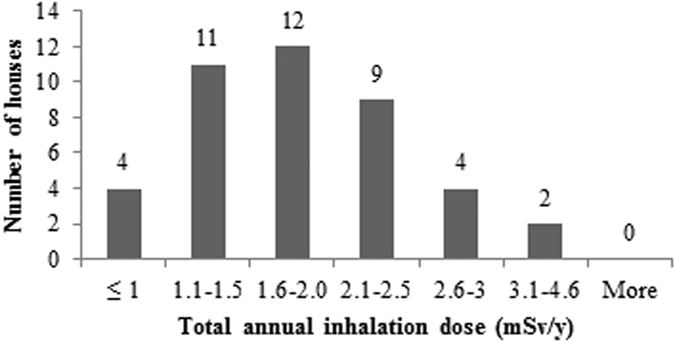
Frequency distribution of total annual inhalation dose in 42 houses of Garhwal Himalaya.

**Figure 18:**
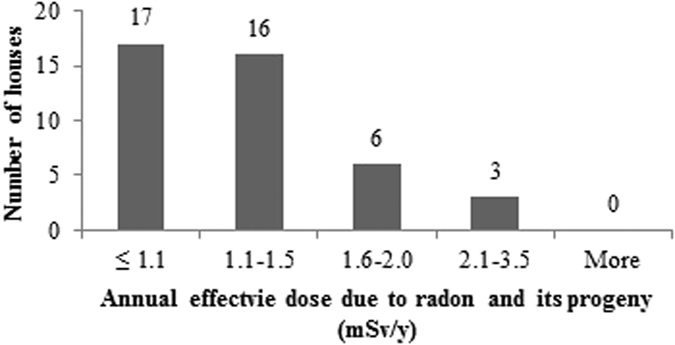
Frequency distribution of annual effective dose due to radon and its progeny in 42 houses of Garhwal Himalaya.

**Figure 19:**
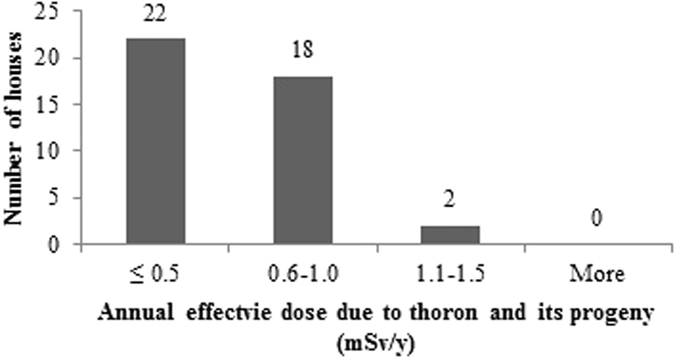
Frequency distribution of annual effective dose due to thoron and its progeny in 42 houses of Garhwal Himalaya.

The frequency distributions of total annual inhalation dose, annual effective dose due to radon and its progeny and annual effective dose due to thoron and its progeny in 42 houses of the study area are represented in the Figs 17, 18, 19. The estimated values of radiation doses have shown no significant health risk due to exposure of radon, thoron and progeny in the study area. Further, it has been seen that the contribution of indoor thoron and its progeny to the total inhalation dose ranges between 13–52% with the mean value of 30%. Thus, thoron cannot be neglected when assessing radiation doses as it was believed in the past.

## Conclusions

The annual average values of radon and thoron concentrations in the study area were found to be somewhat higher than the global average values of 40 Bq/m^3^ and 10 Bq/m^3^ as well as the national average values of 42 Bq/m^3^ and 12.2 Bq/m^3^ of radon and thoron concentrations, respectively. The radon, thoron and progeny concentrations have been found to depend on different seasons of a year. The concentrations of attached and unattached fractions are positively correlated, although only weakly, with Pearson correlations R^2^ = 0.28 and 0.31, for radon and thoron, respectively (significant with p < 0.01 in both cases).

The equilibrium factor for radon and its progeny (0.4) has been found to be in good agreement with its globally assumed value (0.4) and recently calculated value (0.42) for Garhwal Himalaya. However, this factor has been found to be higher than the previously determined value (0.28) of equilibrium factor for radon and its progeny for Garhwal Himalaya. The equilibrium factor for thoron and its progeny (0.07) has been found to be lower than the globally assumed value (0.1) and previously determined value (0.09) for Garhwal Himalaya. However, the estimated value (0.07) of equilibrium factor for thoron and its progeny has been found to be in good agreement with the recently determined value (0.07). It was found that the radon progeny and the equilibrium factor depend largely on the environmental conditions, which may results in the variation in dose calculation. The large variation in measured values of equilibrium factor suggests that while calculating the radiation dose due to the exposure of radon, thoron and their progeny, the equilibrium factors should be determined separately for individual dwelling. This study indicates that the values of equilibrium factors for radon and thoron are reproducible in the study area with recently developed pin-hole dosimeter and DTPS/DRPS techniques.

In general, the radiation doses have shown no significant health risk due to exposure of radon, thoron and progeny in the study area. Moreover, thoron and its progeny have been found to contribute about 1/3^rd^ to the total inhalation dose. This justifies that thoron cannot be neglected when assessing radiation doses as it was believed in the past. However, certain specific houses with very high radon and thoron concentrations need to be investigated in detail with epidemiological survey from health risk point of view. In future, it is planned to perform detail investigation to establish reference radiation level for study area, keeping in view the uncertainties in evaluation of different parameters.

## Additional Information

**How to cite this article**: Ramola, R. C. *et al.* Dose Estimation Derived from the Exposure to Radon, Thoron and their Progeny in the Indoor Environment. *Sci. Rep.* **6**, 31061; doi: 10.1038/srep31061 (2016).

## Figures and Tables

**Table 1:** Descriptive statistics radon, thoron and progeny in 122 houses of study area for rainy season.

	Concentrations (Bq/m^3^)				Estimated Annual Mean Concentrations (Bq/m^3^)			
	Radon	Thoron	Radon Progeny	Thoron Progeny	Radon	Thoron	Radon Progeny	Thoron Progeny
Minimum	4 ± 1	1 ± 0.7	1 ± 0.2	0.1 ± 0.03	6	2	1	0.1
Maximum	198 ± 9	127 ± 8	76 ± 3	7 ± 0.3	265	273	127	9
AM ± SD	41 ± 44	33 ± 30	17 ± 14	2 ± 1	55 ± 59	64 ± 48	23 ± 21	2 ± 1.9
GM	26	22	12	1	35	48	21	1
GSD	3	3	2	3	3	3	2	3
Median	24	23	12	1	32	49	20	1

AM = Arithmetic Mean, SD = Standard Deviation, GM = Geometric Mean, GSD = Geometric Standard Deviation.

**Table 2:** Seasonal variations of radon, thoron, progeny concentrations, unattached fractions and quilibrium factors in 42 houses of Garhwal Himalaya.

	Rainy Season				Winter Season				Summer Season				Annual Mean			
	Min	Max	AM ± SD	GM	Min	Max	AM ± SD	GM	Min	Max	AM ± SD	GM	Min	Max	AM ± SD	GM
Radon Concentration in Bq/m3	11 ± 2	120 ± 7	49 ± 28	42	36 ± 4	182 ± 9	71 ± 32	66	5 ± 1	174 ± 8.7	43 ± 34	34	27	148	54 ± 24	51
EERC (Attached + Unattached) in Bq/m^3^	4.0 ± 0.6	52.2 ± 2.2	16.3 ± 12.1	13.2	6.7 ± 1	65.1 ± 2.5	25.9 ± 14.2	22.6	2.1 ± 0.4	37.6 ± 1.9	15.9 ± 9.7	13.0	7.6	48.6	19.5 ± 8.5	17.9
EERC (Attached) in Bq/m^3^	3.8 ± 0.9	48.8 ± 3.2	15.1 ± 10.3	12.5	5.7 ± 1.1	47.5 ± 3.4	19.2 ± 10	16.7	1.4 ± 0.5	34 ± 2.7	13.6 ± 8.2	11.1	7.0	33.3	16.7 ± 6.3	15.5
EERC (Unattached) in Bq/m3	0.1	15.9	1.2 ± 3.1	0.44	0.2	27.8	6.7 ± 10.7	2.6	0.26	13.0	2.3 ± 2.9	1.3	0.4	17.7	2.8 ± 3.3	1.8
Unattached Fraction (^222^Rn Progeny)	0.01	0.35	0.05 ± 0.06	0.03	0.01	0.84	0.21 ± 0.23	0.11	0.01	0.87	0.16 ± 0.15	0.11	0.02	0.37	0.12 ± 0.07	0.10
F factor for Radon & Progeny	0.10	0.90	0.39 ± 0.23	0.31	0.10	0.91	0.39 ± 0.19	0.34	0.10	0.83	0.41 ± 0.18	0.37	0.10	0.62	0.4 ± 0.13	0.37
Thoron Concentration in Bq/m^3^	3 ± 1	125 ± 8	30 ± 28	22	2 ± 1	210 ± 10	53 ± 52	33	4 ± 1	195 ± 10	47 ± 53	27	5	174	43 ± 37	32
EETC (Attached + Unattached) in Bq/m^3^	0.4 ± 0.1	5.5 ± 0.2	1.9 ± 1.2	1.6	0.3 ± 0.1	4.8 ± 0.2	1.9 ± 1.1	1.6	0.3 ± 0.1	11.2 ± 0.3	1.8 ± 1.9	1.2	0.6	4.6	1.9 ± 1	1.6
EETC (Attached) in Bq/m^3^	0.2 ± 0.1	3.7 ± 0.3	1.4 ± 0.7	1.2	0.3 ± 0.1	4.7 ± 0.3	1.6 ± 0.9	1.4	0.2 ± 0.1	9.6 ± 0.5	1.3 ± 1.6	0.9	0.5	4.1	1.5 ± 0.7	1.3
EETC (Unattached) in Bq/m^3^	BDL	1.8	0.5 ± 0.8	0.2	BDL	1.8	0.3 ± 0.5	0.1	0.1	1.8	0.5 ± 0.5	0.3	BDL	1.5	0.4 ± 0.3	0.3
Unattached Fraction (^220^Rn Progeny)	0.01	0.66	0.21 ± 0.20	0.11	0.01	0.46	0.10 ± 0.10	0.07	0.01	0.70	0.27 ± 0.16	0.21	0.05	0.38	0.19 ± 0.09	0.17
F factor for Thoron & Progeny	0.02	0.36	0.09 ± 0.06	0.07	0.01	0.21	0.06 ± 0.05	0.05	0.01	0.31	0.06 ± 0.05	0.05	0.02	0.17	0.07 ± 0.04	0.06

**Table 3:** Measured Values of Radon and Thoron Concentrations (Bq/m^3^) in different types of houses of Garhwal Himalaya.

	Cemented Houses				Mud Houses				Wooden Houses			
	(Cement + Stone + Bricks)				(Mud + Stones)				(Wood)			
	Ground Floor (Number of Houses = 19)		First Floor (Number of Houses = 21)		Ground Floor (Number of Houses = 10)		First Floor (Number of Houses = 12)		Ground Floor (Number of Houses = 05)		First Floor (Number of Houses = 05)	
	^222^Rn	^220^Rn	^222^Rn	^220^Rn	^222^Rn	^220^Rn	^222^Rn	^220^Rn	^222^Rn	^220^Rn	^222^Rn	^220^Rn
Min	12 ± 2	5 ± 2	5 ± 1	1 ± 0.3	18 ± 3	10 ± 2	6 ± 2	4 ± 1	10 ± 2	15 ± 3	4 ± 1	6 ± 2
Max	123 ± 7	108 ± 8	79 ± 6	122 ± 8	142 ± 8	61 ± 6	74 ± 6	76 ± 6	142 ± 8	61 ± 6	174 ± 12	71 ± 6
Average	56	38	18	22	67	31	21	22	46	42	45	26

**Table 4:** Measured Values of EERC (Bq/m^3^) and EETC (Bq/m^3^) in different types of houses of Garhwal Himalaya.

	Cemented Houses				Mud Houses				Wooden Houses			
	(Cement + Stone + Bricks)				(Mud + Stones)				(Wood)			
	Ground Floor (Number of Houses = 19)		First Floor (Number of Houses = 21)		Ground Floor (Number of Houses = 10)		First Floor (Number of Houses = 12)		Ground Floor (Number of Houses = 05)		First Floor (Number of Houses = 11)	
	EERC	EETC	EERC	EETC	EERC	EETC	EERC	EETC	EERC	EETC	EERC	EETC
Min	6 ± 0.7	0.1 ± 0.03	3 ± 0.5	0.1 ± 0.03	6 ± 0.7	0.3 ± 0.05	3 ± 0.6	0.2 ± 0.04	11 ± 1	0.1 ± 0.03	7 ± 0.8	0.1 ± 0.03
Max	76 ± 3	2 ± 0.1	36 ± 2	3.5 ± 0.2	58 ± 2.3	2.3 ± 0.1	19 ± 1.3	2.8 ± 0.2	51 ± 2	3.6 ± 0.1	39 ± 2	1.2 ± 0.2
Average	19	0.8	14	0.7	28	1.4	9	0.7	21	0.9	18	0.6
